# Understanding the Uniqueness of 2p Elements in Periodic Tables

**DOI:** 10.1002/chem.202003920

**Published:** 2020-11-16

**Authors:** Zhen‐Ling Wang, Han‐Shi Hu, László von Szentpály, Hermann Stoll, Stephan Fritzsche, Pekka Pyykkö, W. H. Eugen Schwarz, Jun Li

**Affiliations:** ^1^ Department of Chemistry & Key Laboratory of Organic Optoelectronics, and Molecular Engineering of the Ministry of Education Tsinghua University Beijing 100084 P.R. China; ^2^ Institut für Theoretische Chemie Universität Stuttgart Stuttgart 70550 Germany; ^3^ Theoretisch-Physikalisches Institut Universität Jena Jena 07743 Germany; ^4^ Department of Chemistry University of Helsinki, POB 55 Helsinki 00014 Finland; ^5^ Theoretische Chemie, Fachbereich Chemie-Biologie Universität Siegen Siegen 57068 Germany; ^6^ Department of Chemistry Southern University of Science and Technology Shenzhen 518055 P.R. China

**Keywords:** bond theory, orbital radii, periodic table, quantum chemistry, radial node effect, sp hybridization

## Abstract

The Periodic Table, and the unique chemical behavior of the first element in a column (group), were discovered simultaneously one and a half centuries ago. Half a century ago, this unique chemistry of the light homologs was correlated to the then available atomic orbital (AO) radii. The radially nodeless 1s, 2p, 3d, 4f valence AOs are particularly compact. The similarity of ***r*** **(2s)≈*r*(2p)** leads to pronounced sp‐hybrid bonding of the light p‐block elements, whereas the heavier p elements with *n*≥3 exhibit ***r*** **(*n*s) ≪** ***r*** **(*n*p)** of approximately **−20 to −30 %**. Herein, a comprehensive physical explanation is presented in terms of *kinetic* radial and angular, as well as *potential* nuclear‐attraction and electron‐screening effects. For hydrogen‐like atoms and all inner shells of the heavy atoms, ***r*** **(2s) ≫** ***r*** **(2p)** by **+20 to +30 %**, whereas ***r*** **(3s)≳*r*(3p)**≳*r*(3d), since in Coulomb potentials radial motion is more radial orbital expanding than angular motion. However, the screening of nuclear attraction by inner core shells is more efficient for s than for p valence shells. The uniqueness of the 2p AO is explained by this differential shielding. Thereby, the present work paves the way for future physical explanations of the 3d, 4f, and 5g cases.

## Graphical abstract

**2p or not 2p?** Similarity of radii (and energies) of the atomic valence orbitals is the precondition for hybridization and hybrid bonding. In the Coulomb potential of H‐like atoms, the 2p orbital without a radial node is much smaller than 2s (2s**≫**2p), whereas higher p orbitals are comparably less contracted (*n*s≳*n*p). The screening of nuclear attraction by the core shells is more effective for the core penetrating s orbitals, so that for the valence orbital radii of ordinary many‐electron atoms of the 2nd row, 2s≈2p, whereas in rows *n*>2, hybridization is less pronounced owing, i.a., to *n*s**≪** *n*p.

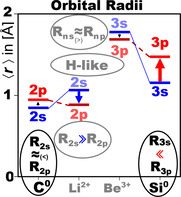

## Introduction

Knowing the trends along a series of related compounds is valuable for every chemist. Understanding the underlying physical reasons is even better. The individual chemical facts can be related to the general physical laws, stepwise, by first finding some generalizing empirical rule, and then rationalizing the rule by atomistic and electronic models that can be deduced from a quantum chemical basis.

The unique chemical behavior of the 2p elements of the second period of the table of elements, in particular of B to F, is well known.[^1^, ^2^, ^3^, ^4^, ^5^, ^6^, ^7^, ^8^, ^9^, ^10^, ^11^, ^12^, ^13^] Yet, this chemical insight still needs better physical rationalization, and better integration into the chemical curricula. We here present a comprehensive analysis of the *n*sp valence atomic orbitals (AOs) of the p‐block elements, that is, of the canonical orbitals from Hartree–Fock or Dirac–Fock or Kohn–Sham levels of theory; which simulate the observable *spatial and energetic* changes in physical ionization and excitation processes.

In 2019, we celebrated the sesquicentenary of the first comprehensive tables of chemical elements, developed by Meyer, Mendeleev, and others in the 1860s.[^1^, ^14^, ^15^] Mendeleev also realized the uniqueness of elements H and Li to F, following earlier notes in Gmelin's handbook of 1843.^[16]^ Although Meyer contributed to the discussion, he did not emphasize the aspects of the “uniqueness”.^[17]^

It took another century until Jørgensen^[2]^ related the uniqueness of the first element in *any* vertical group of the periodic table to the exceptionally small radial extensions of the 1s, 2p, 3d, and 4f AOs. Orbital functions of all atoms had become known through the then‐possible routine atomic structure computations (see also the Supporting Information). The 1s, 2p, 3d and 4f AOs are characterized by having no radial nodes, in contrast to the *nℓ* AOs with higher principal quantum numbers *n*>*ℓ*+1, which have *n*−(*ℓ*+1) radial nodes (*n* and *ℓ* are the principal and angular quantum numbers). This so‐called Radial Node Effect is now linked to a well‐documented set of empirical chemical phenomena.[^2^, ^3^, ^4^, ^5^, ^6^, ^7^, ^8^, ^9^]

## Conceptual Analysis of Observations and Computations

### Radial node effect and core screening

The radii of the valence AOs, the Radial Node Effect, and the screening of the nuclear attraction potential by the electronic core shells were explicated by Shchukarev in great chemical detail, and reviewed in the 1970s.[^3^, ^4^] Following Jørgensen, he was the first to rationalize the comprehensive bulk of empirical chemistry of those elements, where an orbital angular momentum number *ℓ* appears for the first time. Shchukarev named the 1s, 2p, 3d, and 4f AOs kaino(ceno)symmetric (Greek: καινóς, kainos=new) and Pyykkö^[5]^ named them primogenic (Latin: primus=first, genitus=born).

Harris and Jones^[6]^ investigated the different geometric and electronic structures and the bonding in group 14 dimers (C_2_ to Pb_2_) and highlighted the nodelessness of the C 2p shell. In a seminal review, Kutzelnigg^[7]^ pointed out that the distinct hybridization of bonded B, C, N, O atoms is mainly due to the similar radii of their s and p valence AOs, occurring despite the rather different s and p AO energies in the second period. The resulting impressive difference of structure and bonding of C_2_H_2_ and Si_2_H_2_ has recently been elucidated by Ruedenberg et al.^[18]^ Only for F, the very different AO energies *ϵ*(2s)**≪** *ϵ*(2p) suppress any significant hybridization.^[7]^ In recent decades, various excellent, chemically oriented reviews have been published by Kaupp, Huheey, and others.[^8^, ^9^, ^10^, ^11^, ^12^, ^13^] Thereby, the macroscopic chemical observations were realized as empirical trends and qualitatively rationalized at the AO level.

Shchukarev's rationalization was based on *two physical mechanisms*,^[4]^ of potential and of kinetic type, which in cooperation cause the unique pattern of AO radii and thereby yield the unique chemistries of each first element of a group. The first, *potential energy*, mechanism is related to the penetration of the s valence AOs deep into the atomic core, where the attraction of the effective nuclear Coulomb potential is large. This effect of deep potential energy will be quantitatively explored below by an analysis of core screening, using numerical quantum mechanical computations of many‐electron atoms within the orbital model (for details see the Supporting Information, S.2–4). We confirm Shchukarev's educated guess that the p AOs are better shielded from nuclear attraction than the s AOs, meaning an actual deviation from Slater's lowest‐order approximation of similar screening of s and p AOs.^[19]^

Shchukarev was not fully confident about the second mechanism, related to *kinetic energy*, that is, the Radial Node Effect. More radial, instead of more angular, motion means in wave mechanics that the orbitals have more radial maxima and nodes and fewer angular maxima (lobes) and nodes. The nodal pattern of a wave function is determined by the boundary conditions, the stationary energy, and the potential function. This interrelation will be quantitatively explored by using mathematical derivations of one‐electron atoms with different model potentials.[^20^, ^21^] We also elucidate the meaning of the so‐called non‐bonded Pauli repulsions by lower‐energy occupied (as well as virtual unoccupied) orbitals, represented by pseudopotentials^[22]^ that simulate the orbital orthogonality constraint. H and the 2^nd^ period elements have, respectively, no and a particularly small 1s^2^ Pauli‐repelling atomic core shell, as compared to the heavier elements.

The second, kinematic mechanism has become a popular rationalization of the primogenic effect in chemistry, for example, in refs. [5, 8, 9], assuming that the centrifugal force simply causes p AOs to be more expanded than s AOs. However, the chemical differences and respective physical causes appear rather complicated in the four different sets of 1s, 2p, 3d, and 4f elements. Compare, for example, the 1s case (H 1s^1^ vs. Li 2s^1^ & F 2p^5^; and He 1s^2^ vs. Be 2s^2^ & Ne 2p^6^) with the 4f case (the 15 lanthanoids La–Gd and Gd–Lu are chemically similar to Ac and the later actinoids Cm–Lu, whereas the early actinoids Th–Am are more or less akin to d elements Hf–Ir). Clearly, the chemical diversity is richer than expected on the basis of the Radial Node Effect alone.

### The physical problem with the p block

We will elucidate the physical mechanisms that cause the diversity of *n*s–*n*p radii patterns of the chemically diverse light and heavy p‐block elements. The principal quantum number of an AO is [Eq. 1]

**Figure 1:**
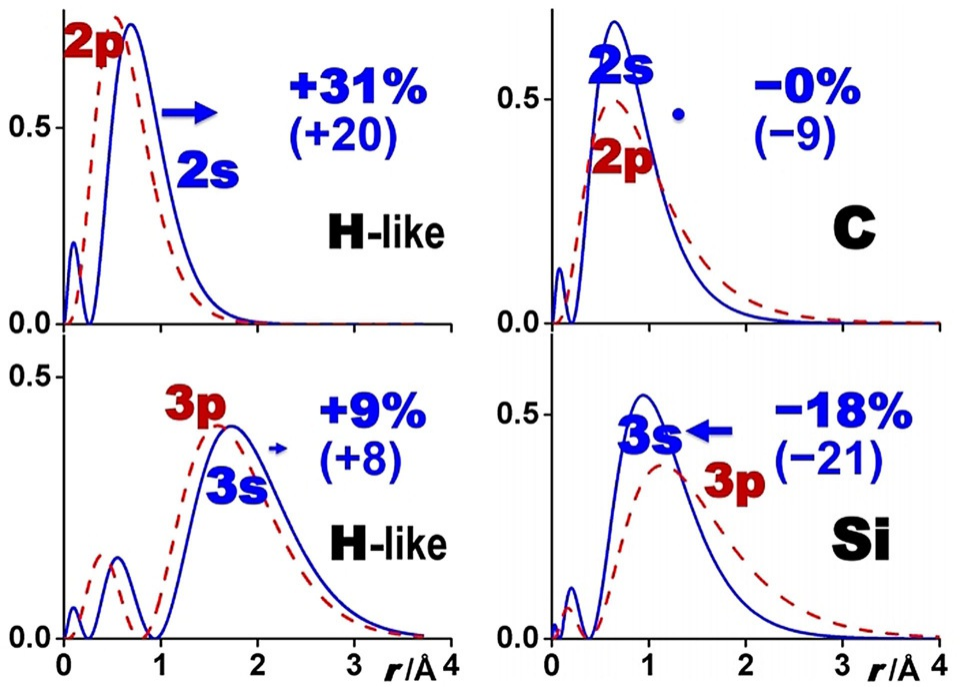
Radial densities *r* ^2^ *φ*(*r*)^2^ of valence AOs *φ* (in atomic units e/Bohr) versus nuclear distance *r* (in Å). The dashed (red in the electronic version) and solid (blue) curves, respectively, refer to p and s AOs. The bold numbers specify the change of orbital radii from p to s in % (referring to the density maxima at *r*_max_; in parentheses for the ⟨*r*⟩ average values; the trends of both are similar and pictured by the bold (blue) arrows). Left: H‐like atoms/ions (here Be^3+^(*nℓ*), without any core shells); 2s is significantly more extended than 2p (ca. +30 and +20 %), whereas 3s and 3p are less different (<+10 %). Right: C and Si: *r*_max_ of C 2s and C 2p are similar, ⟨*r*⟩ of C 2s is a little more compact (ca. −10 %); Si 3s is approximately −20 % smaller than Si 3p.

(1) n=ρ+ℓ+1

where *ρ* is the quantum number of radial nodes, *ℓ* of angular nodes, and the ‘+1’ originates from the Heisenberg Uncertainty principle of quantum theory (see also the Supporting Information, S.6). A common conjecture in chemistry is that the local value of the repulsive centrifugal force for an electron with angular quantum number *ℓ*>0, [Eq. 2]

(2) $$F\left(r\right){}_{\mathrm{centrifugal}}=\ell \left(\ell +1\right)/r{}^{3}$$

(in atomic units, au), moves the outer maximum of an orbital to larger radii. But for hydrogen atoms, s AOs are more extended than p AOs of the same energy (Figure 1, left). The explicit formula for the hydrogenic ⟨*r*⟩ values^[20]^ is (in au; note the minus sign in front of the angular momentum term) [Eq. 3]:

(3) $$\left\langle r\right\rangle =(3\;n{}^{2}-\left(\ell +1\right)\cdot \ell )/2Z$$

On the other hand, for the second period p‐block elements with somewhat larger and slightly screened nuclear Coulomb potentials (by the 1s^2^ core shell), the valence 2s,2p AOs with somewhat different energies *ϵ*(2s)<*ϵ*(2p) have similar radial extensions, *r*(2s)≈*r*(2p), for various definitions of *r* (see Table 2), whereas for the heavier elements of the *n* ^th^ period with significantly larger and significantly more screened nuclear Coulomb potentials (by the 1s^2^ to (*n*−1)p^6^ core shells), the *n*s and *n*p AOs with less different orbital energies *ϵ*(2s)**≲** *ϵ*(2p) have *r*(*n*s)**≪** *r*(*n*p) (Figure 1, right). This pattern is summarized in Table 1. Clearly, both kinetic and potential energy effects need to be considered in any, even qualitative, explanation.

**Table 1:** Pattern of s and p AO radii (see Figure 1, Table 2, and Refs. [20, 23]).

	Hydrogen‐like AOs & core AOs of many‐electron atoms	Valence shell AOs of p‐block atoms
*n=*2	⟨*r*⟩_2s_ **≫**⟨*r*⟩_2p_ (>by +20 to +30 %)	⟨*r*⟩_2s_≈⟨*r*⟩_2p_ (<by 0 to −10 %)
*n*≥3	⟨*r*⟩_*n*s_≈⟨*r*⟩_*n*p_ (>by 0 to +10 %)	⟨*r*⟩_*n*s_ **≪**⟨*r*⟩_*n*p_ (<by −20 to −30 %)

### Differently shaped potentials

At first, we investigate the radial extensions *r*_*nℓ*_ of radial (*ℓ*=0) and angular‐rotational motions (primogenic largest *ℓ*=*n*−1) at a given energy *ϵ* in differently shaped potentials, with the hydrogenic Coulomb potential as the reference. The s/p radii ratios [Eq. 4]

(4) $$Q{}_{n}=\left\langle r\right\rangle {}_{ns}/\left\langle r\right\rangle {}_{np}$$

are for the hydrogenic case *Q*_2_=1.2, *Q*_3_=1.08 (Figure 1, left), *Q*_∞_→1, whereas ⟨*r*⟩_3s_/⟨*r*⟩_3d_=1.29, *Q*_max_=⟨*r*⟩_*n*s_/⟨*r*⟩_*n*,*ℓ*max=*n*−1_→1.5. Clearly, the ratio of hydrogenic *n*s / *nℓ* radii is 1<*r*_*n*s_/*r*_*nℓ*_<1.5. The ‘empirical’ finding is: *Iso‐energetic conversion of radial into angular motion contracts hydrogenic orbitals*.

The hydrogenic AO energies (in a.u.) [Eq. 5]:

(5) $$\epsilon {}_{n\ell }=-Z{}^{2}/2n{}^{2}=-n\cdot \left(Z{}^{2}/2n{}^{3}\right)$$

depend only on the principal quantum number *n*=*ρ*+*ℓ*+1 [Eq. (1)]. The energy quanta of radial and angular motions are equal (see the Supporting Information, S.6). That does not hold for most other potentials such as for the harmonic oscillator, the particle in a box, or the linear potential (modeling, e.g., the strong color interaction of quarks with constant force at large distances, or the vertical motion of a mass on a trampoline). In general, there are large gaps between the s and p orbital levels. For instance, the energies of three‐dimensional harmonic vibrations in potential *V*(*r*)∼+*r* ^*+*2^ (compare with the Coulomb potential *V*(*r*)∼−*r* ^−1^, see Eq. (1), Eq. (5)) are [Eq. 6], [Eq. 7]

(6) $$\epsilon {}_{n\ell }=+n\cdot \left(hv\right)$$

(7) n=2·ρ+ℓ+1.5

In general, one cannot compare quantized s and p states at the same or similar energies, as for atoms. For the harmonic oscillator, *ϵ*(p) is just in the middle between the two corresponding *ϵ*(s) levels.

**Figure 2:**
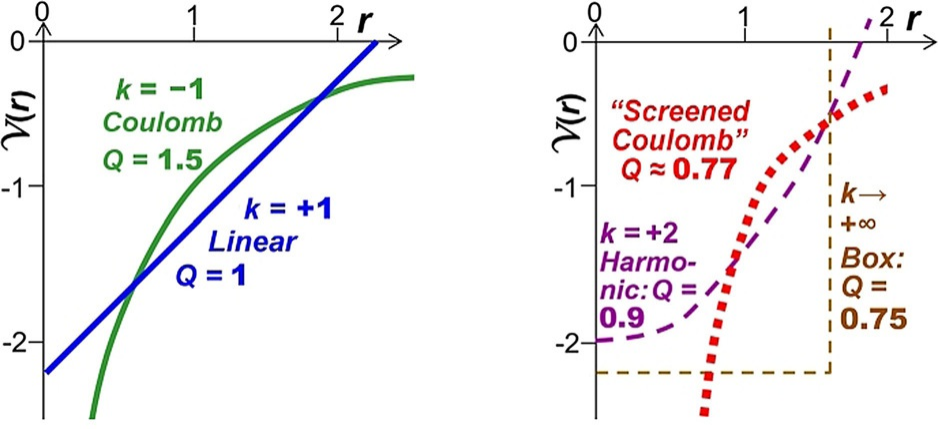
Three‐dimensional spherical potentials *V*(*r*) with different shapes. Left: Green: The electrostatic Coulomb potential ∼−*r* ^−1^ is narrow at short range and flattish at long range, therefore *r*_rad_ **≫** *r*_ang_ and *Q*_max_=*r*_rad_/*r*_ang_>1. Blue: The linear interaction ∼+*r* ^+1^ is the border case with *Q*_max_=1. Right: Potentials are wide at low energies with a steep rise, yielding *r*_rad_ **≪** *r*_ang_ and *Q*_max_<1. Lilac: Harmonic oscillator potential ∼+*r* ^+2^. Red: Typical screened Coulomb potential. Brown: Spherical box potential ∼+*r* ^+∞^. For details, see section S.2b and Table S1 in the Supporting Information.

However, at higher energies, one may compare purely radial (*r*_rad_=*r*_*ℓ*=0_) and angular‐rotational states (*r*_ang_=*r*_*ℓ*‐max_), at similar energies. The semi‐classical approach is a reliable approximation^[21]^ to estimate the ratio *Q*_max_=*r*_rad_ */r*_ang_=*r*_*ℓ*=0_ */r*_*ℓ*‐max_. Some results for potentials *V*(*r*)=*c*⋅*k*⋅*r* ^*k*^ with *k*∈(−2, +∞) show that for *k*∈(−2, +1), the potentials are *flattish at large r* yielding *r*_rad_ > *r*_ang_ (Figure 2, left, *Q*_max_≥1), whereas for *more box‐like* potentials with *k*∈(+1, +∞), *r*_rad_<*r*_ang_ (Figure 2, right, *Q*_max_<1). *Potential wells that are wider at the bottom, and steeper, result in more compact radial motion versus more extended angular motion*.

### Impact of screening on s vs. p valence orbitals

Table 2 displays the ratios Q_*n*_=*r*_*n*s_/*r*_*n*p_ of s over p valence AO radii for p‐block atoms. *Q_n_* values <1 indicate smaller s than p AOs. The ratio values scatter by only a few percent over a period in the p block. Throughout, the ratios of the s/p valence orbital radii are reduced from hydrogen to atoms with core shells by Δ*Q_n_*∈[−0.30, −0.23], remarkably independent of the cores: 1s^2^ for 2sp, or (1s2s2p)^10^ for 3sp, or (1s–3d)^28^ for 4sp, or (1s–4d)^46^ for 5sp. The radius patterns of H‐like or inner‐core AOs are contrasted with the valence AOs of the p‐block elements in Table 1.

**Table 2:** Ratios *Q_n_*=*r*_*n*s_/r_np_ of the various valence orbital radii (*r*_max_ , ⟨*r*⟩, √⟨*r* ^2^⟩) in period *n*. Results for H from Bethe.^[20]^ Dirac–Fock results for many‐electron atoms from Desclaux.^[23]^ Δ*Q_n_* is the mean reduction of the hydrogenic *Q_n_* value. Different computational approaches yield very similar trends for *r*_max_ , ⟨*r*⟩, and √⟨*r* ^2^⟩, for the whole p‐block.[^7^, ^8^, ^9^, ^23^, ^25^, ^26^, ^27^]

*n*	*r*_2s_/*r*_2p_	H	B	C	N	O	F	Mean	Δ*Q*_2_
2	*r*_max_	1.309	0.96	1.00	1.03	1.05	1.06	1.02	−0.29
	⟨*r*⟩	1.200	0.90	0.91	0.92	0.92	0.92	0.91	−0.29
	√⟨*r* ^2^⟩	1.183	0.87	0.88	0.89	0.89	0.89	0.88	−0.30
									
	*r*_3s_/*r*_3p_	H	AI	Si	P	S	CI	Mean	Δ*Q*_3_
3	*r*_max_	1.090	0.78	0.82	0.86	0.88	0.89	0.85	−0.25
	⟨*r*⟩	1.080	0.76	0.79	0.81	0.83	0.84	0.81	−0.27
	√⟨*r* ^2^⟩	1.072	0.75	0.78	0.80	0.82	0.83	0.80	−0.28
									
	*r*_4s_/*r*_4p_	H	Ga	Ge	As	Se	Br	Mean	Δ*Q*_4_
4	*r*_max_	1.044	0.75	0.79	0.81	0.84	0.86	0.81	−0.24
	⟨*r*⟩	1.043	0.72	0.75	0.78	0.80	0.81	0.77	−0.27
	√⟨*r* ^2^⟩	1.04	0.71	0.75	0.77	0.79	0.80	0.76	−0.28
									
	*r*_5s_/*r*_5p_	H	In	Sn	Sb	Te	I	Mean	Δ*Q*_5_
5	*r*_max_	1.03	0.75	0.79	0.82	0.84	0.85	0.81	−0.23
	⟨*r*⟩	1.027	0.72	0.76	0.78	0.80	0.81	0.78	−0.25
	√⟨*r* ^2^⟩	1.025	0.72	0.75	0.78	0.79	0.81	0.77	−0.26

We now investigate how the shielding of the nuclear attraction of the outer valence s and p AOs by the inner core shells emerges. Different relativistic Dirac–Fock and Kohn–Sham density functional calculations yield similar trends. Technical details are given in the Supporting Information (S.4). We increase the nuclear charge of an excited H atom and simultaneously add electrons.

**Figure 3:**
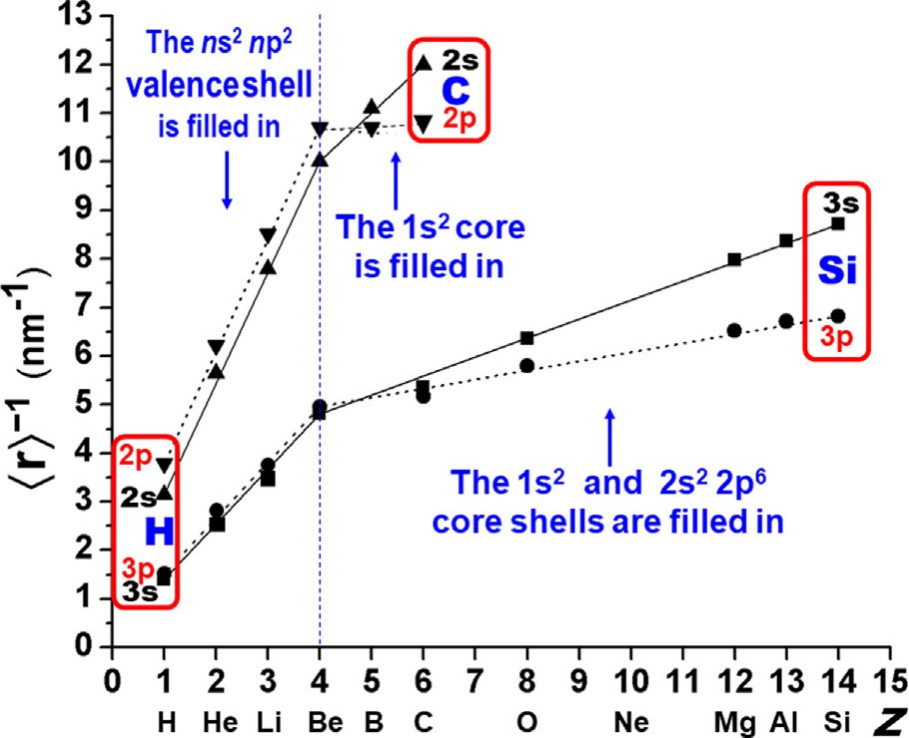
Inverse radii ⟨*nℓ*|*r*|*nℓ*⟩^−1^ (in nm^−1^) corresponding to *Z*_eff_ of the atomic *nℓ* valence orbitals versus nuclear charge number *Z* (with electronic core–hole configurations (see Supporting Information, S.4**–**5). The straight lines (full for s, dotted for p) guide the eyes from H* *n*(sp)^1^ through Be** *n*s^2^ *n*p^2^ to C 1s^2^–2s^2^2p^2^ or, respectively, Si 1s^2^2s^2^2p^6^–3s^2^3p^2^. The smaller slopes for *n*p versus the steeper for *n*s from Be onward to C or Si show that the *n*p valence orbitals are better shielded from the (increasing) nuclear charge by the (increasing number of) core electrons than the *n*s orbitals. Note the change of order from H* and Be** (*r*_s_>*r*_p_) to C or Si (*r*_s_<*r*_p_).

First, we add valence electrons to H (*n*s,*n*p)^1^ (H*), obtaining Be *n*s^2^ *n*p^2^ (Be**) with an empty core and populated valence shells as in atoms C (*n=*2), Si (*n=*3), or Ge (*n=*4). Since the AO radii vary as ∼1/*Z* (compare Eq. (3)), we plot 1/⟨*r*⟩ versus *Z* to obtain nearly linear lines (Figure 3). Up to an *n*‐dependent scale factor, 1/⟨*r*⟩ had been defined as *Z*_eff_ by Hartree,^[24]^ that is, Figure 3 actually shows *Z*_eff_ versus *Z*. The steep slopes on the left side indicate strong AO contraction and *Z*_eff_ increase upon increase of *Z*, owing to the weak screening of the increasing nuclear charge when electrons are added in the same valence shell. This is in accordance with Slater's^[19b]^ medium–small mean shielding constant with an average value of *σ*=0.35 for s,p shells. The screening constant *σ* corrects the nuclear charge *Z* to *Z*−*σ*; this can be applied to the exponent in the wavefunction^[21]^ or to expressions for orbital energies, or for orbital radii, as suggested by Hartree. He stressed “there is no single ‘screening parameter’ which will represent all the properties. This is perhaps not always sufficiently realized”. In quantum defect theory, two weakly varying parameters *σ*_*ℓ*_ and *δ*_*ℓ*_ are needed to describe an outer orbital with quantum numbers *n*,*ℓ*, the effective screening by *Z*−*σ*_*ℓ*_, and the effective phase shift or quantum number by *n*−*δ*_*ℓ*_.

Second, we populate the core shells until reaching C, Si, or Ge. Screening by inner shells is more efficient (*σ*→1), whereas screening by outer (Rydberg) shells would be even weaker (*σ*→0). The radii of the sp valence shells behave approximately as expected for Slater's nuclear screening by the next inner (*σ*_s_=*σ*_p_=0.85) and further in‐bound sp shells (*σ*_s_=*σ*_p_=1), corresponding to the flat lines on the right side of Figure 3. However, somewhat different screening for s than for p AOs was already noticed by Clementi et al.^[25]^ This is here reconfirmed by the steeper lines for *n*s than for *n*p AOs in Figure 3. This is due to the stronger core penetration of the s AOs. Concerning the AO radii, the differential screening of the valence s versus p AOs by the next inner sp core shell is better described by *σ*_s_≈0.7 and *σ*_p_≈0.9 to 1.0, than by Slater's single averaged value of *σ*_s,p_≈0.85. As a consequence, the valence s AOs of heavier p‐block atoms are eventually more contracted than the p AOs.

### Core vs. valence screenings

**Figure 4:**
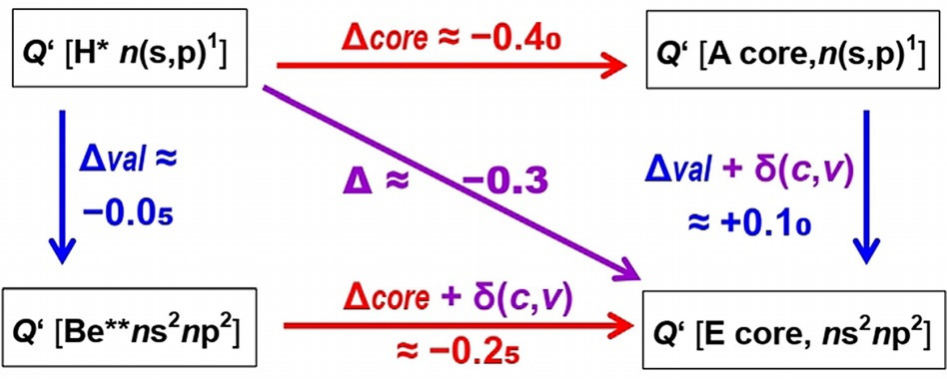
Variation of the ratios *Q_n_*=*r*_*n*s_/*r*_*n*p_ of valence orbital radii, upon strong differential core–(s,p) valence inter‐shell screening (Δ_core_≈−0.40, Red); upon weak (s,p) valence intra‐shell screening (Δ_val_≈−0.05, Blue); and the double‐screening cross term of opposite sign (*δ*(c,v)≈+0.15, Lilac).

The s/p radii ratios *Q_n_* for 1‐electronic H*, for 4‐valence electronic Be**, both with empty lower shells, and for 1‐valence electronic atoms with filled core shells (A=Li, Na, Cu), and for group 14 atoms of the second to fourth period (E=C, Si, and Ge) are displayed in Table 3. The ⟨*r*⟩ and *r*_max_ ratios show similar trends as sketched in Figure 4. The core‐valence inter‐shell shielding of nuclear attraction of the valence *n*s versus *n*p AOs by the (1s)^2^, (1s–2p)^10^, and (1s–3d)^28^ core shells reduces the hydrogenic *r*_*n*s_/*r*_*n*p_ ratio *Q_n_* throughout by approximately −40 %, whereas the s^2^p^2^ intra‐valence shell shielding is nearly an order of magnitude smaller (ca. −5 %). The two shielding effects interfere and damp each other (by ca. +15 %), which is not uncommon for two different ‘perturbations’. The joint screening reduction of *Q_n_* then sums up to approximately −40 %−5 %+15 %≈−30 %, throughout, as mentioned above (Table 2).

**Table 3:** Ratios *Q_n_* of *n*s/*n*p valence orbital radii of excited hydrogen‐like states H* (*n*s,*n*p)^1^, of group 1 or 11 atoms A (*n*s,*n*p)^1^, of highly excited empty‐core Be** *n*s^2^ *n*p^2^ configurations, and of atoms E *n*s^2^ *n*p^2^, E=C, Si, and Ge.

Atom	Valence state^[a]^	Atomic core	Comput. method^[b]^	⟨*r*⟩_*n*s_/ ⟨*r*⟩_*n*p_	⟨*r*_max_⟩_*n*s_/ ⟨*r*_max_⟩_*n*p_
H*	2s^1^ or 2p^1^	1s^0^	“exact”	1.20	1.31
Li		1s^2^	KS‐STO	0.80	0.82
					
Be**	2s^2^2p^2^	1s^0^	KS‐num.	1.09	1.235
C		1s2	various	0.90(2)	1.01(1)
					
H*	3s^1^ or 3p^1^	1s^0^2s^0^2p^0^	“exact”	1.08	1.09
Na		1s^2^2s^2^2p^6^	KS‐STO	0.70	0.68
					
Be**	3s^2^3p^2^	1s^0^2s^0^2p^0^	KS‐num.	1.05	1.07
Si		1s^2^2s^2^2p^6^	various	0.78(1)	0.82(1)
					
H*	4s^1^ or 4p^1^	(1s–3d)^0^	“exact”	1.043	1.044
Cu		(1s–3d)^28^	KS‐STO	0.65	0.68
					
Be**	4s^2^4p^2^	(1s–3d)^0^	KS‐num.	1.023	1.034
Ge		(1s–3d)^28^	various	0.75(1)	0.79(1)

[a] Configuration state averaged. [b] “Exact”=explicit nonrelativistic point‐charge solutions by Bethe.^[20]^ KS‐STO=nonrelativistic Kohn–Sham PBE approach with STO basis, using the ADF program of Baerends, see the Supporting Information, S.3a. KS‐num.=numerical relativistic Dirac–Kohn–Sham–Slater approach by Fritzsche (see the Supporting Information, S.4b).^[28]^ Various=Average of Kohn–Sham, Hartree–Fock, or Dirac–Fock approaches; the number in parentheses is the standard deviation (see also the Supporting Information, S.5).

## Inferences

When periodic tables of elements were designed 150 years ago, it was realized that the first element in a column is special. A good century later, a large body of observed facts on the light homologs had been collected, classified, and related to the comparatively small radii of the 1s, 2p, 3d, and 4f valence AOs, resulting in different bonding schemes for the first versus the heavier homologs. Concerning the unique p elements of the second period, another half century passed until the final step of understanding is now achieved. The physical elucidation reveals why *r*_*n*s_ **≪** *r*_*n*p_ for the valence shells of most p‐block elements (with dominant p‐bonding), except *r*_2s_≈*r*_2p_ in the second period (with dominant sp‐hybrid bonding). The hydrogenic relations are in contrast *r*_2s_≫*r*_2p_, and *r*_*n*s_≳*r*_*n*p_ for *n*>2. Although independent‐electron orbitals in principle do not exist by themselves in many‐electron systems,^[29]^ they have proven as an approximate and very useful concept and tool to explain and understand the behavior of chemical systems. In the present context, several important points need to be taken into account:

(1) The orbital set of an atom (or molecule) emerges as a coherent set, describing the *quantized motions* of electrons in the nuclear Coulomb *potential*, *screened* by the other electrons. Canonical orbitals are conveniently chosen as a mutually orthogonal set. Owing to the *mutual* orthogonality and to the atomic potential *V*(*r*), the inner node positions of different radial orbitals of given *ℓ* occur at similar places, determined by the shape of the potential. The number of radial and angular orbital nodes and extrema is the number of quanta of the respective radial and angular motions. The number of quanta is required either by the Pauli principle and the occupied core shells, or by electronic excitation into a higher orbital above unoccupied ones. An orthogonality constraint on lower (occupied or unoccupied) AOs can be simulated by a pseudopotential, which is repulsive in the inner region. A pseudopotential is a useful tool for computations and for explanations, it does not represent a physical causal effect of mutual orthogonality.

(2) Coulomb potentials ∼−*r* ^−1^ are flattish at larger nuclear distances, with a narrow deep well at the center. This potential shape yields rather large orbital radii of the 2s, 3s, and 4s AOs, that is, significantly larger than the radii of orbitals 2p, 3d, 4f at the same energies. Radial motion (nodes) in a nuclear Coulomb potential moves the outer maximum of an orbital to larger radii (the Radial Node Effect) than the centrifugal force, in particular in the case of the 2s–2p orbital pair, where the 2p AO has no radial node. The radially nodeless 7i (*ℓ*=6) AO is smaller than 7s and even than 6s. For the 2s,2p AOs of H as well as of heavy atoms with occupied core shells, (*r*_max_)_2s_/(*r*_max_)_2p_ is around 1.3, and ⟨*r*⟩_2s_/⟨*r*⟩_2p_ is around 1.2.

(3) Radial oscillation samples the potential at large distances as well as in the nuclear vicinity, especially for the *ℓ*=0 s states. For atomic ions *Z* ^+*q*^, the effective *Z*_eff_ varies from *Z*_eff_=*Z* at *r*=0 to *Z*_eff_=1+*q* at large *r*. The difference of the effective potential inside the atomic cores, compared with pure Coulomb potentials, leads to contracted s AOs (the Core Screening Effect). Slater's lowest‐order approximation of similar core screening for s and p valence AOs is not accurate enough for the chemical problems at hand. Although a basically Coulombic potential yields the orders ⟨*r*⟩_2s_ **≫**⟨*r*⟩_2p_ and ⟨*r*⟩_3s_≳⟨*r*⟩_3p_≳⟨*r*⟩_3d_ for the inner core orbitals of all heavier atoms (the Radial Node Effect, acting for *n=*2), the outer valence orbitals in the screened Coulomb potential follow the inverted order ⟨*r*⟩_2s_≲⟨*r*⟩_2p_, but ⟨*r*⟩_3s_ **≪**⟨*r*⟩_3p_<⟨*r*⟩_3d_ (the Core Screening Effect, shifting all ratios *Q_n_* for *n*≥2 by ca. −0.3).

(4) Some update of chemical explanations appears appropriate. (a) The centrifugal force owing to a quantum of angular motion (corresponding to an angular node of the AO), is comparable to the expanding effect of a quantum of radial motion, represented by a radial node. Which is more effective depends on the shape of the effective potential in the core. (b) Electrons in valence s AOs of many‐electron atoms are less shielded and more attracted and contracted than their p counterparts. (c) Both the energetic *and* radial patterns of the valence AOs determine the bonding behavior of an element.

Other aspects may also be highlighted,[^30^, ^31^] in particular the diverse Pauli repulsions by the divers atomic core shells,[^13c^, ^36^] namely the small 1s^2^ core of the 2^nd^ period and the ‘standard sized’ (*n*‐1)p^6^ or (*n*‐1)p^6^d^10^ cores of the heavier *n* ^th^ periods codetermine the interatomic separations, and thereby the different valence‐orbital overlaps. More exotic core interpretations (such as by spurious nodes and outer tails of inner core orbitals, or by taking formal charges seriously) have been refuted.[^32a^, ^32b^]

## Conclusion

Kinetic *and* potential energy effects *and* their interplay need be analyzed together in physical explanations of chemistry. There is a tendency to explain covalent bonding electrostatically, whereas the electronic–kinetic aspect is physically dominating.^[33]^ Conversely, the radii ratio of the s/p valence AOs governing the bonding and chemistry of the p‐block elements is dominantly determined by the screening of the electrostatic core potential, whereas the kinetic Radial Node Effect has more pedagogic appeal.

The relevance of each term (radial vs. angular motion in a more or less shielded Coulomb potential) can only be judged on the basis of quantitative data, in particular in the more complicated cases of d and f orbitals. That is needed for a better future understanding of chemistry over the periodic table. Investigations of differential screening connected to the Radial Node Effect, as presented here for the p‐block, are still awaiting their turn to trace the physical origin of the chemical peculiarities of the 3d, and 4f, not to mention the hypothetical 5g block.^[34]^ The peculiarity of the non‐primogenic early 5f elements belongs to this field, too.

In summary, the 2p elements are known to be qualitative‐chemically different from their heavier congeners. The physical origin is the quantitative interplay of the electronic kinetic and potential energies of the valence orbitals: 2p has no radial node and little radial kinetic energy, thus 2p is radially contracted. All s orbitals are weakly shielded from nuclear attraction, thus 2s, 3s, 4s etc. are radially contracted. Therefore *r*_2s_/*r*_2p_≈1, but *r_ns_*/*r_np_*<1 for *n*>2. The uniqueness of the 2p (as well as the 3d and 4f) block elements exhibiting the quantum primogenic effect plays a significant role in general chemistry. The effect is also essential for the topical support influence in heterogeneous catalysis.^[35]^

## Conflict of interest

The authors declare no conflict of interest.

## Supporting information

As a service to our authors and readers, this journal provides supporting information supplied by the authors. Such materials are peer reviewed and may be re‐organized for online delivery, but are not copy‐edited or typeset. Technical support issues arising from supporting information (other than missing files) should be addressed to the authors.

Supplementary
