# Supplementary material for: Understanding the Uniqueness of 2p Elements in Periodic Tables
Source: Chemistry. 2020 Nov 16;26(67):15558–64. doi: 10.1002/chem.202003920 (PMC7756678; doi:10.1002/chem.202003920)
Supplement: Supplementary file 1 — Supplementary [file CHEM-26-15558-s001.pdf]

Chemistry–A European Journal

Supporting Information

# Understanding the Uniqueness of 2p Elements in Periodic Tables

Zhen-Ling Wang,[a] Han-Shi Hu,[a] László von Szentpály,[b] Hermann Stoll,[b]
Stephan Fritzsche,[c] Pekka Pyykkö,*[d] W. H. Eugen Schwarz,*[a, e] and Jun Li*[a, f]

# Contents

Text S.1: Potential versus Kinetic Energies of Valence Electrons and the Virial Theorem p. 2

Text S.2: The Classical Model and the Semi-Classical Quantum Approach

a. Classical Model with Bohr-Sommerfeld Constraints (*Figure S1*. Hydrogenic Bohr-Sommerfeld Orbits) p. 4

b. Semiclassical Quantum Approach of Jeffreys, Wentzel, Kramers and Brillouin (*Figure S2*. Semiclassical Trajectories for Hydrogen ; *Table S1*. Semiclassical Radii Ratios) p. 5

Text S.3: Jørgensen and Shchukarëv on 1s-, 2p-, 3d-, 4f-Elements

a. Jørgensen on d-Metal Complexes p. 7

b. Shchukarev (Щукарёв) on Secondary Periodicity p. 8

Text S.4: Some Computational Details and Results

a. Nonrelativistic Basis-Set Density-Functional Approximation (*Table S2*. Atomic Valence Orbitals; *Table S3*. Points of Maximum Radial Density) p. 9

b. Relativistic Numerical Density Functional Approximation (*Table S4*. Valence Shells Above Core Holes) p. 10

Text S.5: Notes on Core-Hole Configurations and Configuration Averages p.12

Text S.6: Radial-Angular Partitioning of the Kinetic Orbital Energy p.13

Text S.7: Orbital Energies of Alkali-Metal and Noble-Gas Pseudo-Atoms (*Figure S3*) p.14

S.8 Additional References p.15

## S.1 Potential vs. Kinetic Energies of Valence Electrons and the Virial Theorem

When the charge and energy density distributions of a molecular system have been determined experimentally or theoretically, one can describe them in detail, for instance by applying the "Quantum Theory of Atoms in Molecules" (QTAIM).[S1] In addition to the question of 'what' is, one can also ask 'why' it is so, applying various approaches such as the "Energy Decomposition Analysis" (EDA),[S2] the "Natural Bond Orbital" (NBO) analysis" [S3] or the "Quasi-Atomic Bonding Analysis" (QUABA).[S4-S6] They show that the binding of electrons in atoms and of atoms in molecules (chemical bonding) is due to the competition of "potential pull" versus "kinetic pressure" of the electrons, both changing during the binding process. Their ratio in variationally optimized stationary states is related to the Coulombic virial theorem.[33a] We comment on three points: potential versus kinetic energy, the relevance of the general virial theorem, and the radial nodes of valence orbitals.

(i) Chemical matter, as applied and investigated by us humans, is usually in a quasi-stationary state, governed by the *equilibrium of kinetic and potential energies* (local energy densities $\mathcal{T}(r)$ and $\mathcal{V}(r)$, and observable total expectation values $\langle\mathcal{T}\rangle$ and $\langle\mathcal{V}\rangle$, respectively), their sum forming the Hamiltonian $\mathcal{H} = \mathcal{T} + \mathcal{V}$. In common quantum-chemical atomic units (au) and for the nonrelativistic case, $\mathcal{T}(\mathrm{r}) = -1/2\mathrm{M} \cdot \nabla^2\varphi / \varphi$,[a)] where M means the mass of the particle (M = 1 for the electron); and $\mathcal{V} = -\mathrm{Ze}^2/r$ for an electron in the field of a nucleus of charge +Ze (e = 1), for example. Any physical rationalization of material phenomena in chemistry must consider the *kinetic and potential energies and their interplay*. A typical example for the importance of kinetic energy is found in the mechanism of chemical bonding, where the exclusive confinement to potential energy aspects is missing the main point of covalent electron sharing and relative delocalization.[33, S4-S6]

(ii) For the expectation values of the total kinetic and potential energies, $\langle\mathcal{T}\rangle$ and $\langle\mathcal{V}\rangle$, of a bound stationary state of many particles, the nonrelativistic virial theorem,

$$2\langle\mathcal{T}\rangle = \langle\mathbf{r} \cdot \nabla\mathcal{V}\rangle \ , \qquad \text{(S1.1)}$$

takes a particularly simple form, if all particles interact by a power potential,

$$\mathcal{V}(\mathrm{r}) = \zeta \cdot k \cdot r^k \quad (k > -2;\ \zeta > 0)\ , \qquad \text{(S1.2)}$$

namely for total energy $\langle\mathcal{H}\rangle = E$,

$$\langle\mathcal{V}\rangle = 2/(2+k) \cdot E\ , \quad \langle\mathcal{T}\rangle = k/(2+k) \cdot E\ , \quad \langle\mathcal{V}\rangle / \langle\mathcal{T}\rangle = 2/k\ . \qquad \text{(S1.3)}$$

For the common Coulombic case ($k = -1$), this yields

$$\langle\mathcal{V}\rangle = 2E\ , \quad \langle\mathcal{T}\rangle = -E\ , \quad \langle\mathcal{V}\rangle / \langle\mathcal{T}\rangle = -2\ , \qquad \text{(S1.4)}$$

which holds for all-electron ab-initio approaches of sufficient accuracy. For pure angular motion without radial motion (except the Heisenberg uncertainty contributions) in a centrosymmetric potential, the orbit(al) radius is then

$$r_{\mathrm{ang}} \approx \sqrt[k]{\frac{2E}{\zeta k(2+k)}}\ . \qquad \text{(S1.5)}$$

---

a) The Bader school [S1] prefers $\mathcal{T}_{mod}(\mathrm{r}) = +1/2\mathrm{M} \cdot |\nabla\varphi|^2 / |\varphi|^2$, which is positive everywhere like the classical kinetic energy and yields the correct observable value upon integration, but is different locally and cannot be directly applied in the common Hamiltonian or Lagrangean equations to determine the wavefunction of the matter-field.

For the H atom, this gives $r_{ang} \approx (\ell+1)(\ell+1) / Z$ (in au), while the exact quantum mechanical value is $r_{ang} \approx (\ell+1)(\ell+1.5) / Z$ , being large by ~ $1/2\ell \rightarrow 0$ for large quantum number.

(iii) In chemistry, valence-only approaches are common, in particular for large and heavy systems, either replacing the atomic cores by pseudopotentials,[22, S7] or keeping the valence shells orthogonal to the frozen atomic cores.[S2, S8] Both approaches have been used here. The shapes of the outer maxima of the valence orbitals, the ratios of the valence orbital radii, and the chemical bonding due to the orbital overlaps from the different approaches are found to be very similar. However, in the pseudopotential approach, a part of the radial *kinetic* energy is replaced by pseudo-*potential* energy of the valence shell, whereby the total binding energies are kept constant, but the pseudo-virial ratio increases,[33b] while the opposite usually occurs in the frozen core approach:

$$\langle \mathcal{V}_{\text{val-pseudo}}\rangle / \langle \mathcal{T}_{\text{val-pseudo}}\rangle < -2 \; ; \qquad \langle \mathcal{V}_{\text{val-frocore}}\rangle / \langle \mathcal{T}_{\text{val-frocore}}\rangle > -2 \, . \qquad \text{(S1.6)}$$

The reason for this change is that in wave mechanics, the wave-lengths and nodes determine the momenta and the kinetic energy density ($-\frac{1}{2M}\nabla^2 \varphi / \varphi$). If energetically lower AOs of same angular momentum are *occupied*, the Pauli principle (electronic Fermi-Dirac statistics) may require an increased radial motion of electrons in orbitals with additional radial nodes. This part of the kinetic energy density may be replaced by a repulsive pseudopotential (simulating the Pauli repulsion by the lower occupied shells). Thereby the effective kinetic energy value is decreased, and the potential energy value becomes less negative by the same amount. The Pauli repulsion replaces the effects due to higher radial quantum number and more radial inner nodes. The orthogonality of an orbital for an optically excited state to lower *empty* orbitals may also be mimicked by a repulsive pseudopotential, which in this case would not include the electrostatic repulsion by occupied orbitals.

# S.2 Classical Model and Semi-Classical Quantum Approach

## *S.2a Classical Model with the Bohr-Sommerfeld Constraints*

In classical (Newtonian) mechanics, the motion of a particle in a centrosymmetric potential may be classified as *angular*-rotational (on a circle, without net radial momentum, "$\ell_{max}$"), as *radial*-oscillatory (on a linear trajectory, with zero angular momentum, "$\ell = 0$"), or as mixed radial-oscillatory and angular-rotational (on an ellipsis). In the limit of nonrelativistic electrostatic physics, the electro-magnetic coupling is infinitely weak,[S9] the emission of radiation is quenched and the electronic orbits are stable.[b)] When Bohr developed his model of one-electron H-like atoms,[S10] he ad hoc added the quantization of energy or angular momentum as a constraint upon classical mechanics, in order to reproduce the experimental spectral energies, thereby also reproducing reasonable values for the sizes of the atomic orbits. Sommerfeld explicitly extended Bohr's circular model to include also elliptical orbits.[S11]

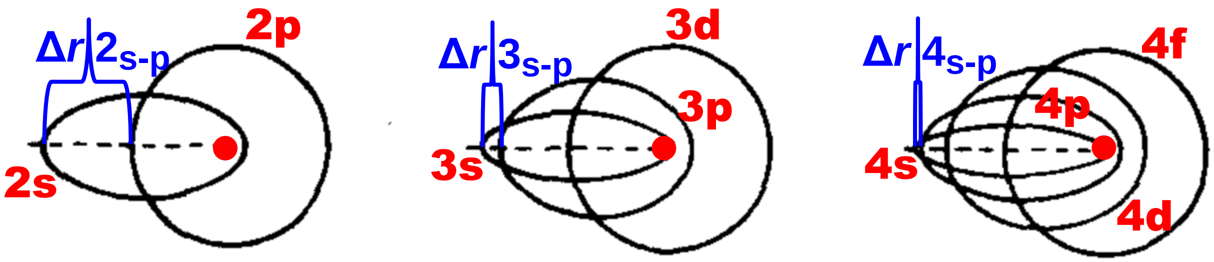


***Figure S1***. Classical Hydrogenic Bohr-Sommerfeld Orbits (from Fig.4 of Sommerfeld, 1916.[S11] The red dots are here added to indicate the nuclei. The blue $\boldsymbol{\Delta r}$ $\boldsymbol{n_{s-p}}$ indicate the *relative* differences of outer turning points of the *n*s and *n*p orbits. It shows: $r_{2s} / r_{2p} > 1$, while for large *n*: $r_{ns} / r_{np} \rightarrow 1$, but $r_{ns} / r_{n,\ell max} \rightarrow 1.5$, as in the quantum mechanical case. Note that the outer parts of ellipses have a larger weight, since the particle is slower there.

The classical Kepler Orbits for a given energy have all the same large diameter and frequency. The orbiting particle is slow on the outer parts of an eccentric ellipsis, and therefore this part contributes more to observables such as the mean nuclear distance. Sommerfeld's graphic, reproduced in Figure S1, highlights three important aspects correctly: (i) at given energy, s-type orbits are more extended than p-type orbits; (ii) the relative s/p difference decreases for increasing quantum number; (iii) however, for increasing quantum number, the average radii ratio increases for orbitals with smallest and largest angular momentum.

Yet it must be admitted that the classical Bohr-Sommerfeld model has some serious defects. It describes the 3-dimensional atoms as flat 2-dimensional objects, and it does not consider any states with zero angular-momentum. Therefore the semi-classical approach will be exploited.

---

[b)] As long as the central attraction is weaker than ~ $-1/r^2$, i.e. of type $k{\cdot}r^k$, $k > -2$, the position, momentum and energy densities remain integrable along the line from turning-points $r_{min}$ to $r_{max}$ in the effective potential at both the non-relativistic and relativistic approximations.

### S.2b Semiclassical Quantum Approach of Jeffreys, Wentzel, Kramers and Brillouin

A better semi-classical simulation of s states is by a *linear* rotationless ($\ell = 0$) radial motion,[S12-S20] which may be chosen as the limit of the elliptical Kepler orbits ($\ell \rightarrow +0$), where the electron would oscillate between $r = 0$ (at the nucleus) and $r = 2 \cdot R_o$ (see Figure 2). The latter is twice as far from the nucleus as for the *circular* orbit of same energy. Since the electron is slow at the outer turning point and fast near the nucleus, the time-averaged nuclear distance is larger than the arithmetic mean of the turning-point distances, $(0+2)/2 \cdot R_o = 1 \cdot R_o$, the integration giving $R_{\leftrightarrow} = 1.5 \cdot R_o$. One might also choose the $\ell$=0 radial oscillation from $+2R_o$ through the nucleus to $-2R_o$, which gives the same averaged nuclear distance of $R_{\leftrightarrow} = 1.5 \cdot R_o$.

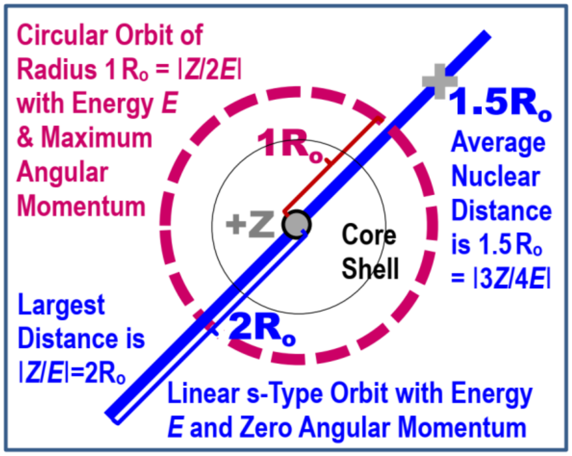


***Figure S2***. Semiclassical Trajectories for Hydrogenic s and $\ell_{max}$ electrons, moving non-relativistically at energy $E$ in the field of a point-like Coulomb charge $+Ze$, for vanishing electromagnetic coupling.
*Dashed red circle*: Angular-rotational motion with maximum angular momentum, corresponding to quantum-mechanical 2p, 3d, 4f, … primogenic/kainosymmetric orbitals ($\ell_{max} = n-1$; angular-rotational radius $r_{ang} = R_o = Z/2E$ au; 1 au ≈ 0.529 Å).
*Full blue line*: Motion at same energy without any rotation, i.e. radial oscillation to and away from the nucleus, corresponding to s orbitals. Because of the 'flatness' of the Coulomb potential $\sim -1/r$ at large $r$, the classical outer turning points of 'slow' motion are at large nuclear distances of $2 \cdot R_o$, yielding a time-averaged distance of $r_{rad} = 1.5 \cdot R_o$.

***Table S1.*** Semiclassical radii ratios $Q = r_{rad} / r_{ang}$ .

| $k$ | Potential | $Q = r_{rad} / r_{ang}$ |
|---|---|---|
| −2 | 'Gulping Hole' | $\rightarrow \infty$ |
| $-^3/_2$ | | ≈ 1.937 |
| −1 | Electric Coulomb attraction | $^3/_2 = 1.5$ |
| −½ | | $^{35}/_{27} \approx 1.296$ |
| → 0 | Constant potential | → 1.166 |
| ½ | | $^{15}/_{14} \approx 1.071$ |
| 1 | Linear potential | 1 |
| $^3/_2$ | | ≈ 0.944 |
| 2 | Spherical harmonic oscillator | $\sqrt{8} / \pi \approx 0.900$ |
| 3 | | ≈ 0.835 |
| | Spherical box | $^3/_4 = 0.75$ |

Concerning the general power potential $\mathcal{V}(\mathrm{r}) = \zeta \cdot k \cdot r^k$ for a heavy-light particle system, the radius for pure angular motion from the classical virial theorem, eq. (S1.5), or equivalently from the lowest order semi-classical quantum approach, is

$$k \cdot \zeta \cdot r_{\mathrm{ang}}{}^{k} = 2E/(2+k) \,. \qquad \text{(S2.1)}$$

The lowest-order semi-classical value for the time-averaged radius of pure radial motion (without Langer correction) is obtained by [21, S15, S17]

$$r_{\mathrm{rad}} = \int \mathrm{dr} \bullet \mathrm{r} \,/\, \sqrt{[\langle \mathcal{H} \rangle - \mathcal{V}(r)]} \quad \Big/ \quad \int \mathrm{dr} \,/\, \sqrt{[\langle \mathcal{H} \rangle - \mathcal{V}(r)]} \,,$$

which yields the ratios $Q_{\mathrm{max}} = r_{\mathrm{rad}} / r_{\mathrm{ang}}$ displayed in Table S1, used for Figure 2.

In summary, hydrogenic classical Bohr-Sommerfeld orbits as well as semi-classical or quantum-mechanical orbitals with maximum angular momentum and without any motion in radial direction (in addition to the Heisenberg uncertainty effects) of primogenic kainosymmetric 2p, 3d, 4f, etc. type are more compact than rotationless states of s type at same energy. However, if the potential is wider and deeper at small distances from the center (spherical harmonic oscillator, effective potential of an atom with many occupied core shells, a spherical box), the s-states become ‘sucked in’ with comparatively small average radii.

# S.3 Jørgensen and Shchukarëv on 1s-, 2p-, 3d-, 4f-Elements

## *S.3a Jørgensen on d-Metal Complexes and Oxidation States of s, p, d, f Valence Shells*

In his Ph.D. dissertation of 1957 on ‘Energy Levels of Complexes and Gaseous Ions’,[2a] Jørgensen discussed ligand field theory and noted the difference of 3d *vs.* *n*d and 4f *vs.* 5f ions. A dozen years later, in 1969 just a century after Mendeleev’s famous publication, Jørgensen developed in his book on ‘Oxidation Numbers and Oxidation States’ a correct perception and clear understanding of the order of orbital energies and radii of free atoms and of atoms in compounds,[2b] which is rarely presented in chemistry textbooks. We here repeat his ‘chemically relevant ***valence orbital energy*** order’ from his eq. (3.11),

$$1s \ll 2s<2p \ll 3s<3p \ll 3d<4s<4p \ll 4d<5s< 5p \ll 4f<5d<6s<6p \ll 5f < 6d<7s< \ldots \quad (S2.3)$$

We stress two points: (i) Jørgensen explicitly distinguishes *orbital energy gaps* which are chemically small (i.e. where different AOs can be mixed or hybridized by the ligand fields), from those which are chemically large. The latter, indicated by $\ll$, introduce the periodicity of the chemical elements. (ii) The displayed *orbital energy order*, in particular 3d<4s etc., is the correct order of the valence AOs for the whole system of chemical elements – except for the heavier members of the first groups (K, Rb, Cs, Fr; Ca, Sr, Ba, Ra; La, Ac, Th), and except for negatively charged metal ions in complexes, where *n*s < (*n*-1)d, (*n*-2)f holds. Yet, nearly all textbooks teach the so-called (*n*+ℓ,*n*)-rule,[S21-S23] which does not distinguish between chemically different gap sizes, and does not apply to the majority of elements in the f and d and heavy p blocks.[S23-,S25]

Concerning the ***valence orbital radii***, Jørgensen noted that 3d and 4s are not that different in energy, but 3d becomes significantly smaller than 4s for the transition metal ions in their common formal oxidation states. Another important insight is that ‘the first *n*-value for each given ℓ-value, 1s, 2p, 3d and 4f, is characterized by much smaller radii than the subsequent *n*ℓ groups’,[2b: p.49] and he continues that ‘This generalization is *only remotely connected with the fact that these orbitals have no radial nodes*’, meaning that the quantum kinematics is not the main cause for the small size of the primogenic orbitals. Jørgensen was also aware of the long known (*n*-1)d and (*n*-2)f valence orbital radial collapse, reviewed by Connerade.[S26]

We have shown in the main text, that the fact of similar radii of the 2s valence orbitals and the ‘primogenic’ 2p orbitals is the combined result of (1) differential nuclear shielding, contracting the s orbitals more than the p orbitals, and (2) that the ‘first *n*-value for given ℓ-value’ means that there is no radial excitation for 2p. While in the textbooks it is convincingly argued that angular rotation acts more expanding than radial oscillation, just the opposite is true for an atomic Coulomb potential that is only slowly varying at larger nuclear distances. Pure rotation means that there is no radial excitation (radial excitation quantum number is $\rho = 0$), which also means in wave theory that there is no radial wave node.

*In summary*, there is a relation between a radial node surface and the radial extension. The comparable radial extensions of 2s and 2p are the result of two opposite effects on 2s, the radial node expansion of the 2s, and the radial contraction of 2s due to its deep core penetration that leads to strong nuclear attraction and weaker nuclear shielding. The better nuclear shielding of 2p than of 2s depends on the occupation of the core shells, while the radial node effect expanding 2s more than 2p is not dependent of the core shell occupation. The latter effect depends on the quantum number, which is either limited from below by the Pauli principle in case of occupied core shells, or determined by radial excitation, e.g. due to photon absorption. We admit that the realistic pattern of physical interrelations is somewhat complex.

### *S.3b Shchukarëv on Secondary Periodicity and on Nodeless s, p, d, f Orbitals*

In 1915 Biron published his observation that the elemental properties vary in a zigzag manner down a column of the periodic table. He baptized this phenomenon 'secondary periodicity'. The secondary periodicity is more pronounced at the end than at the beginning of a period, because the main reason of the secondary periodicity is the period lengthening in double steps due to the population of the d and f shells, causing the respective scandide and lanthanide contractions, relatively most pronounced in groups 4 and the following ones. The secondary periodicity is also more pronounced at the top than at the bottom of the periodic table, because at the bottom the atomic orbital density of states increases and also becomes perturbed by relativistic effects. Shchukarëv understood the importance of various extents of screening of the nuclear attraction potential by the various types of atomic core shells.[3,4] Like Jørgensen, he was aware of the comparably small radii of orbitals without radial nodes, but was not sure about the importance of the kinetic radial node effect.

# S.4 Some Computational Details and Results

## *S.4a Nonrelativistic Basis-Set Density-Functional Approximation*

We have performed average-configuration self-consistent-field Kohn-Sham calculations of ground, valence-excited and core-excited configurations (by fixing the occupation numbers of the inner shells) of He, Li, Be, B, C, O, Na, Mg, Al, Si, K, Rb and Ge atoms. The PBE density-functional approximation was applied.[S27] We used triple-zeta Slater-type basis sets [S28] with even-tempered exponents (factors around 1.5 to 1.6); the common basis sets are not useful for the core-hole states. The ADF-2018 software was applied.[S8] The chosen configurations and some results of orbital energies $\boldsymbol{\varepsilon}_{n\ell}$ and orbital radii $\boldsymbol{r}_{n\ell} = \langle\varphi_{n\ell}|\, r\, |\varphi_{n\ell}\rangle$ are displayed in Tables S2 and S3, and plotted in Figure 3 of the main text. For comparison, some analytic results for H* are shown, they are not contaminated by self-interaction, finite basis and continuum coupling errors.

***Table S2.*** Atomic valence orbital energies ($\varepsilon$ in eV) and radii (***r*** in pm).[a)]

| **Atomic Configuration** | | $\boldsymbol{\varepsilon_{2s}}$ | $\boldsymbol{\varepsilon_{2p}}$ | | $\boldsymbol{r_{2s}}$ | $\boldsymbol{r_{2p}}$ | $\boldsymbol{r_s/r_p}$ |
|---|---|---|---|---|---|---|---|
| C-$1s^2 2s^2 2p^2$ | | **-13.8** | **-5.3** | | **83.3** | **95.3** | **0.874** |
| B-$\mathbf{1s^1}2s^2 2p^2$ | | **-12.0** | **-5.9** | | **90.0** | **93.1** | **0.967** |
| Be-$\mathbf{1s^0}2s^2 2p^2$ | | **-10.2** | **-6.5** | | **99.7** | **93.2** | **1.070** |
| Li-$\mathbf{1s^0 2s^{1.5} 2p^{1.5}}$ | | **-6.8** | **-4.7** | | **128.** | **118.** | **1.085** |
| He-$\mathbf{1s^0 2s^1 2p^1}$ | | **-4.1** | **-3.2** | | **178.** | **161.** | **1.106** |
| H-$\mathbf{2s^{½} 2p^{½}}$ | | **-3.40** | **-3.40** | | **318.** | **265.** | **1.200** |
| | | | | | | | |
| **Atomic Configuration** | | $\boldsymbol{\varepsilon_{3s}}$ | $\boldsymbol{\varepsilon_{3p}}$ | | $\boldsymbol{r_{3s}}$ | $\boldsymbol{r_{3p}}$ | $\boldsymbol{r_s/r_p}$ |
| Si-$1s^2 2s^2 2p^6 3s^2 3p^2$ | | **-10.8** | **-4.1** | | **115** | **147** | **0.782** |
| Al-$1s^2 2s^2 \mathbf{2p^5} 3s^2 3p^2$ | | **-10.2** | **-4.1** | | **119** | **149** | **0.799** |
| Al-$1s^2 \mathbf{2s^1} 2p^6 3s^2 3p^2$ | | **-10.2** | **-4.1** | | **120** | **149** | **0.805** |
| Mg-$1s^2 \mathbf{2s^0} 2p^6 3s^2 3p^2$ | | **-9.6** | **-4.1** | | **125** | **153** | **0.817** |
| O-$1s^2 2s^2 \mathbf{2p^0} 3s^2 3p^2$ | | **-7.2** | **-4.1** | | **157** | **173** | **0.908** |
| C-$1s^2 \mathbf{2s^0 2p^0} 3s^2 3p^2$ | | **-5.7** | **-3.9** | | **187** | **193** | **0.969** |
| Be-$\mathbf{1s^0 2s^0 2p^0} 3s^2 3p^2$ | | **-4.8** | **-4.1** | | **208** | **202** | **1.030** |
| H-$\mathbf{3s^{½} 3p^{½}}$ | | **-1.51** | **-1.51** | | **714** | **661** | **1.080** |

[a)] Differences between the numbers in Figs. S1 and S3 are due to the differences between basis KS-PBE vs. numerical DF-S approaches.

***Table S3***. Maximum radial-densities points $r_{\max}$ (in pm) of $D(r) = r^2 \cdot \varphi(r)^2$ of atomic core- and valence-orbitals φ, of neutral H, C and Si atoms, and ratios of core over valence orbital radii, and %-ages by which the valence p orbitals are smaller or bigger than the valence s orbitals.[a]

| | | | | |
|---|---|---|---|---|
| H(1s): 52.9 | H(2p): 211.7 | | C(1s): 9.2 | Si(2p): 20.7 |
| $r_{1s}/r_{2s}$ = **0.19** | $r_{2p}/r_{3s}$ = **0.31** | | $r_{1s}/r_{2s}$ = **0.14** | $r_{2p}/r_{3s}$ = **0.22** |
| H(2s): 277.1 | H(3s): 691.9 | | C(2s): 64.6 | Si(3s): 95.2 |
| H(2p): 211.7 | H(3p): 635.0 | | C(2p): 64.4 | Si(3p): 115.2 |
| $r_{2s}/r_{2p}$ −1: **−24%** | $r_{3s}/r_{3p}$ −1: **−8.2%** | | $r_{2s}/r_{2p}$ −1: −0.25**%** | $r_{3s}/r_{3p}$ −1: **+21%** |

[a] C and Si: Dirac-Fock results of 1973.[23]

## S.4b Relativistic Numerical Density Functional Approximation

The core-hole states have been computed with a program package using numerical integration which is practically exact, also fulfilling the boundary conditions, namely the JAC tools.[28] The orbitals are generated in the relativistic Dirac-Fock-Slater mean-field potential and represented in a B-spline basis. Respective orbital energies and orbital radii (in terms of $r_{max}$, $\langle r \rangle$ and $\sqrt{\langle r^2 \rangle}$ ) and the $r_s/r_p$ radii ratios are displayed in Table S4 for the higher excited empty-core states of He, Li and Be in comparison to the ordinary states of the alkali atoms Li, Na, K and Rb. Analytical orbital radii ratios for excited hydrogen-like states are displayed for comparison. We note that the empty-core *nsn*p states of He, Li and Be have radii ratios $r_s/r_p > 1$, qualitatively similar to the hydrogen-like $r_{ns} / r_{np}$ ratios.

***Table S4***. Open Valence Shells Above Core Holes (configuration averages). He-($n$sp)$^2$, Li-($n$sp)$^3$, and Be-($n$sp)$^4$, for $n$ = 2 to 4 (continuum resonances of the neutral atoms with empty inner shells). Orbital energies ε (in eV) ; radii $r_{max}$, $\langle r \rangle$ and $\sqrt{\langle r^2 \rangle}$ (in pm) ; radii ratios $r_s/r_p$ ($p_{ave}$ means weighted average of $p_{1/2}$ and $p_{3/2}$): obtained by the Dirac-Fock-Slater (DFS) configuration-average approach with numerical integration. For comparison, the excited $n$s, $n$p states of H*, and the ordinary $n$s, $n$p states of the alkali atoms [a)] are also displayed.

| **2s2p shell** | | | | |
|---|---|---|---|---|
| H* 1s$^0$**(2s,2p)$^1$** | −ε | $r_{max}$ | $\langle r \rangle$ | $\sqrt{\langle r^2 \rangle}$ |
| ε, $r_{2s} / r_{2p}$ (exact)<br>(DFS) | 3.401<br>3.401 | 1.309<br>1.307 | 1.2<br>1.200 | 1.183<br>1.183 |
| He** 1s$^0$**2s$^1$2p$^1$** | −ε | $r_{max}$ | $\langle r \rangle$ | $\sqrt{\langle r^2 \rangle}$ |
| $2s_{½}$ | 3.20 | 155 | 191 | 208 |
| $2p_{ave}$ | 2.38 | 126 | 171 | 191 |
| $r_{2s} / r_{2p}$ | | 1.23 | 1.12 | 1.09 |
| Li** 1s$^0$**2s$^1$2p$^2$** | −ε | $r_{max}$ | $\langle r \rangle$ | $\sqrt{\langle r^2 \rangle}$ |
| $2s_{½}$ | 5.58 | 107 | 135 | 148 |
| $2p_{ave}$ | 3.75 | 86 | 122 | 138 |
| $r_{2s} / r_{2p}$ | | 1.23 | 1.11 | 1.07 |
| Be** 1s$^0$**2s$^2$2p$^2$** | −ε / eV | $r_{max}$ | $\langle r \rangle$ | $\sqrt{\langle r^2 \rangle}$ |
| $2s_{½}$ | 8.82 | 79 | 103 | 113 |
| $2p_{ave}$ | 5.51 | 64 | 94 | 107 |
| $r_{2s} / r_{2p}$ | | 1.23 | 1.09 | 1.05 |
| Li 1s$^2$**(2s,2p)$^1$** [a)] | −ε / eV | $r_{max}$ | $\langle r \rangle$ | $\sqrt{\langle r^2 \rangle}$ |
| $2s_{½}$ | 2.15 | 172 | 224 | 246 |
| $2p_{ave}$ | 0.63 | 230 | 324 | 365 |
| $r_{2s} / r_{2p}$ | | 0.74$_5$ | 0.69 | 0.67 |

(continued)

| **3s3p shell** | | | | |
|---|---|---|---|---|
| H* $1s^0$**(3s,3p)$^1$** | −ε | $r_{max}$ | ⟨$r$⟩ | √⟨$r^2$⟩ |
| ε, $r_{3s}$ / $r_{3p}$ (exact)<br>(DFS) | 1.512<br>1.511 | 1.090<br>1.084 | 1.080<br>1.078 | 1.072<br>1.070 |
| He** $1s^0 2s^0 2p^0$**$3s^1 3p^1$** | −ε | $r_{max}$ | ⟨$r$⟩ | √⟨$r^2$⟩ |
| $3s_{½}$ | 1.42 | 381 | 410 | 438 |
| $3p_{ave}$ | 1.28 | 355 | 392 | 420 |
| $r_{3s}$ / $r_{3p}$ | | 1.07 | 1.05 | 1.04 |
| Li** $1s^0 2s^0 2p^0$**$3s^1 3p^2$** | −ε | $r_{max}$ | ⟨$r$⟩ | √⟨$r^2$⟩ |
| $3s_{½}$ | 2.52 | 258 | 287 | 307 |
| $3p_{ave}$ | 2.22 | 240 | 273 | 290 |
| $r_{3s}$ / $r_{3p}$ | | 1.07 | 1.05 | 1.06 |
| Be** $1s^0 2s^0 2p^0$**$3s^2 3p^2$** | −ε / eV | $r_{max}$ | ⟨$r$⟩ | √⟨$r^2$⟩ |
| $3s_{½}$ | 3.88 | 195 | 220 | 235 |
| $3p_{ave}$ | 3.29 | 182 | 210 | 226 |
| $r_{3s}$ / $r_{3p}$ | | 1.07 | 1.05 | 1.04 |
| Na $1s^2 2s^2 2p^6$**(3s,3p)$^1$** [a)] | −ε / eV | $r_{max}$ | ⟨$r$⟩ | √⟨$r^2$⟩ |
| $3s_{½}$ | 2.87 | 168 | 215 | 234 |
| $3p_{ave}$ | 0.83 | 269 | 344 | 377 |
| $r_{3s}$ / $r_{3p}$ | | 0.63 | 0.62 | 0.62 |

| **4s4p (and 5s5p) shell** | | | | |
|---|---|---|---|---|
| H* $1s^0$**(4s,4p)$^1$**(exact) | −ε | $r_{max}$ | ⟨$r$⟩ | √⟨$r^2$⟩ |
| ε, $r_{4s}$ / $r_{4p}$ | 0.850 | 1.044 | 1.043 | 1.039 |
| Be** (1s2sp3spd)$^0$**$4s^2 4p^2$** | −ε | $r_{max}$ | ⟨$r$⟩ | √⟨$r^2$⟩ |
| $4s_{½}$ | 2.16 | 366 | 382 | 405 |
| $4p_{ave}$ | 1.98 | 355 | 373 | 397 |
| $r_{4s}$ / $r_{4p}$ | | 1.03 | 1.02 | 1.02 |
| K $1s^2$ to $3s^2 3p^6$**(4s4p)$^1$** [a)] | −ε | $r_{max}$ | ⟨$r$⟩ | √⟨$r^2$⟩ |
| $4s_{½}$ | 2.37 | 212 | 262 | 283 |
| $4p_{ave}$ | 0.83 | 315 | 391 | 424 |
| $r_{4s}$ / $r_{4p}$ | | 0.67 | 0.67 | 0.67 |
| Rb $1s^2$ to $3d^{10} 4s^2 4p^6$**(5s5p)$^1$** [a)] | −ε | $r_{max}$ | ⟨$r$⟩ | √⟨$r^2$⟩ |
| $5s_{½}$ | 2.32 | 222 | 274 | 294 |
| $5p_{ave}$ | 0.81 | 339 | 415 | 448 |
| $r_{5s}$ / $r_{5p}$ | | 0.66 | 0.66 | 0.66 |

[a)] The simple Slater density functional without a self-interaction correction such as the Latter correction yields too softly bonded and diffuse atomic valence shells of the free alkali metal atoms.

## S.5 Notes on Core-Hole Configurations and Configuration Averages

We have investigated two occupation-paths from the one-electronic $ns^1$ and $np^1$ states of the H atom to the ground states of the many-electronic atoms of group 14 (C,Si, Ge) with valence configurations $ns^2np^2$ in the $n^{th}$ row of the periodic table. Either the core shells are filled at first, and then the three remaining 'valence electrons' are added, or vice versa, at first the $ns^2np^2$ valence configuration is formed, and then the electrons are filled into the unoccupied core shells. If the change of some electronic property of the valence shell due to screening of the nuclear attraction by the other electrons is denoted by $\Delta_{screening}$, it may be generally partitioned into three contributions, due to inter-shell screening by the core electrons, due to intra-shell screening by the other three valence electrons, and due to the change of screening due to core-valence interactions:

$$\Delta_{screening} = \Delta_{core\text{-}screening} + \Delta_{valence\text{-}screening} + \Delta_{core\text{-}valence\text{-}interaction\text{-}screening}$$

Highly excited states and respective configuration averages with holes in the inner atomic shells are coupled to, and less or more dissolved in the continuum. The interaction between the formally localized core-hole states and the ionization continua of the many lower states may lead to a comparatively sharp Feshbach resonance, or respectively, to a broad shape resonance. Further, there is strong core-valence correlation. For example, a hole at the bottom of a core band, such as $2s^12p^6$, strongly interacts with the $2s^22p^5$ core hole via valence shake up, for instance Al-$1s^22s^12p^63s^23p^2$ becomes 'dissolved' in the manifold of many tens of Al-$1s^22s^22p^5$(3s3p3d 4s4p4d4f etc.)$^4$ and Al-$1s^22s^22p^6$(3s3p3d 4s4p4d4f etc.)$^3$ states of same symmetry.

Yet we may introduce core-hole determinants as model constructs between two types of reasonably well defined states: (i) the spectroscopically well observable and more or less sharp excited one-electron states of hydrogen with empty lower orbitals ('core orbitals') and a single 'valence electron' in a higher $n$s or $n$p orbital, and (ii) the well observable sharp many-electron ground-states of Li, Na, Cu and C, Si, Ge with filled core shells and 1 or 4 valence electrons. The hypothetical intermediate constructs Be** with $ns^2np^2$ valence configuration above empty cores shells are then calculated to understand and explain, how and why the orbital energies and radii change from the excited one-electron hydrogen case to the many-electron atomic ground-state.

It is common in chemistry textbooks to list the experimental ionization potentials, i.e. the ground state energy differences of relativistic orbit-orbit and spin-orbit coupled *SLJ* states, of chemically unbound atoms in physical vacuum, and declare these data as prototypical for chemically bound atoms in chemical compounds. Obviously this is a bit misleading. Due to the lower symmetry of the molecular ligand field as compared to nearly vanishing perturbations in physical vacuum, the *SLJ* couplings and splittings are largely quenched in 'chemical atoms in molecules'. In chemistry the configurationally averaged properties of atoms are more relevant. For instance, in the cases of C and Si, the properties of the free-atomic ${}^3P_0{}^e$ ground states are chemically less important than the $s^2p^2$ (and $sp^3$) valence configurational averages. Therefore, the latter are displayed in Table S1.

# S.6 Radial-Angular Partitioning of the Kinetic Orbital Energy

In atomic units, the quantum kinetic energy per orbital is given by $\int d\boldsymbol{r}^3 \cdot D_{kin}(\boldsymbol{r})\ \varphi(r)^2$ . The kinetic energy density of a one-electronic wave field (orbital φ) is given by $D_{kin}(\boldsymbol{r}) = \mathcal{T}\circ\varphi(\boldsymbol{r}) / \varphi(\boldsymbol{r})$. The kinetic energy operator in Cartesian coordinates is $\mathcal{T} = -½\cdot(d^2/dx^2 + d^2/dy^2 + d^2/dz^2)$. In an atomic central potential, a partitioning in spherical coordinates is more appropriate,

$$\mathcal{T} = \mathcal{T}_{\mathbf{rad}} + \mathcal{T}_{\mathbf{ang}} = [-½\cdot d^2/dr^2 - 1/r\cdot d/dr] + [\boldsymbol{\ell}^2 / 2r^2] , \quad \text{(S6.1)}$$

where atomic units (a.u.) are assumed, the electronic mass $m_e = 1$, and $\boldsymbol{\ell}$ means the angular momentum operator, which may be replaced for the (nonrelativistic) eigenfunctions in radial coordinates by $\boldsymbol{\ell}^2\circ \rightarrow \ell(\ell+1)\cdot$.

With hydrogen-like orbitals $\varphi(Z;n\ell m)$ for nuclear charges Z = 1,2,3,…, angular momentum quantum numbers ℓ = 0,1,2,…, radial excitation quantum numbers ρ = 0,1,2,…, orientational (magnetic) quantum numbers $m$, and principal quantum numbers

$$n = \rho + \ell + 1, \quad \text{(S6.2)}$$

one obtains the expectation values of the radial and angular kinetic energies as

$$\varepsilon_{\mathbf{kin,rad+}} = [\rho + \delta] \cdot Z^2 / 2n^3 \quad \text{and} \quad \varepsilon_{\mathbf{kin,ang+}} = [\ell + (1-\delta)] \cdot Z^2 / 2n^3 , \quad \text{(S6.3)}$$

$$\delta = (\ell+1)/(2\ell+1) \quad \text{and} \quad (1-\delta) = \ell/(2\ell+1) . \quad \text{(S6.4)}$$

The terms δ and (1−δ) are, respectively, 1 and 0 for s-orbitals, ⅔ and ⅓ for p-orbitals, $^3/_5$ and $^2/_5$ for d-orbitals, and $^4/_7$ and $^3/_7$ for f-orbitals.

For the case without any radial and angular excitation, ρ = ℓ = 0 and n = 1, i.e. for the 1s orbital, we have only the (nonrelativistic) zero-point 'Zitterbewegung' due to the Heisenberg Uncertainty Principle, which contributes through δ = 1 to $\varepsilon_{\mathbf{kin,rad}}$ the amount of $\varepsilon_{\mathbf{Heis}} = +½\cdot Z^2$ a.u. (eqs. S5.3,4). For additional radial motion ρ > 0 (i.e. $n$s orbitals with $n \geq 2$), $\varepsilon_{\mathbf{kin}} = \varepsilon_{\mathbf{kin,rad+}} = [\rho+1]\cdot Z^2/2n^3$, where $+1\cdot Z^2/2n^3$ a.u. is the (radial) Heisenberg Uncertainty contribution. For large ℓ and large rotation energy, δ and (1−δ) both approach ½, i.e. the total Heisenberg Uncertainty term $\varepsilon_{\mathbf{Heis.}} = Z^2/2n^3$ a.u. contributes equally to both $\varepsilon_{\mathbf{kin,rad+}} = (\rho+½)\cdot Z^2/2n^3$ and $\varepsilon_{\mathbf{kin,ang+}} = (\ell+½)\cdot Z^2/2n^3$.

We may then interpret the hydrogen-like orbital kinetic-energy formula (see S2.1)

$$\varepsilon_{\mathbf{kin}} = +(\rho + \ell + 1) \cdot Z^2/2n^3 \quad \text{(S6.5)}$$

as containing three contributions, the radial energy quanta $\rho\cdot Z^2/2n^3$, the angular energy quanta $\ell\cdot Z^2/2n^3$, and a Heisenberg Uncertainty contribution of $Z^2/2n^3$, which is of dominantly radial type for states where the kinematics is of dominantly radial type, but which contributes equally to radial and angular motion in the case of large angular momentum.

We note that the energies of the harmonic oscillator are $2\varepsilon_{\mathbf{pot}} = 2\varepsilon_{\mathbf{kin}} = \varepsilon = n \cdot h\nu$ with

$$n = 2\rho + \ell + 1.5 . \quad \text{(S6.6)}$$

The lowest d and second s states have same energy, their radius ratio is Q = 15/16 = 0.9375.

## S.7 Further Supporting Numerical Results

Figure S3 displays the orbital energies of $ns^1$ valence orbitals in atoms with different cores. The lowest line in lilac displays the cases of the ordinary ground states of the alkali metal atoms, Li-2s, Na-3s, K-4s, Rb-5s, Cs-6s. We note the secondary periodicity down the group. We compare that with the cases, where the K-shell is ‘absorbed’ by the nucleus (green line), meaning that the nuclear charge is reduced by 2 units with $1s^0$ core (i.e. Li-$1s^22s^1$ → H*-$1s^02s^1$ ; Na-$1s^22s^22p^63s^1$ → F*-$1s^02s^22p^63s^1$ ; etc. ; Cs-$1s^22s^2\cdots5p^66s^1$ → I*-$1s^02s^2\cdots5p^66s^1$).

The core-penetrating *n*s valence orbitals are partially shielded from nuclear attraction by electrons in the 1s core shell. This effect decreases with increasing *n*, since the *n*s density in the K-shell decreases by nearly an order of magnitude for each step of *n*; the shielding is most pronounced for ε(2s) of H*-$2s^1$ to Li-$1s^22s^1$. **The two 2s orbitals have the same nodal pattern, but feel different potentials**: Coulombic in the first case, where ε(2s) = ε(2p) and r(2s) > r(2p), screened Coulombic in the second case with stronger attraction at small r, where ε(2s) < ε(2p) (and r(2s) > r(2p), compare Tables S1 and S4). The 2s is more sensitive to core-shell screening than the 2p, since the 2p is largely kept out of the K-shell by the centrifugal force. Similar trends as for ε hold for the orbital radii (compare Figure 3).

When both the $1s^2$ and $2s^2$ shells are absorbed by the nucleus (upper blue line), we see a large stabilization of 3s from N*-$1s^02s^02p^63s^1$ to Na-$1s^22s^22p^63s^1$, since 3s has significant density in the next inner-more L-shell (namely: smaller step of ε(3s) from N*-**$1s^02s^0$**$2p^63s^1$ to F*-**$1s^22s^0$**$2p^63s^1$, larger step to Na-**$1s^22s^2$**$2p^63s^1$; or: larger step of ε(3s) from N*-**$1s^02s^0$**$2p^63s^1$ to F*-**$1s^02s^2$**$2p^63s^1$, larger step to Na-**$1s^22s^2$**$2p^63s^1$). Again the *n*s orbital in various environments has same nodal structure, but feels differently screened Coulomb potentials.

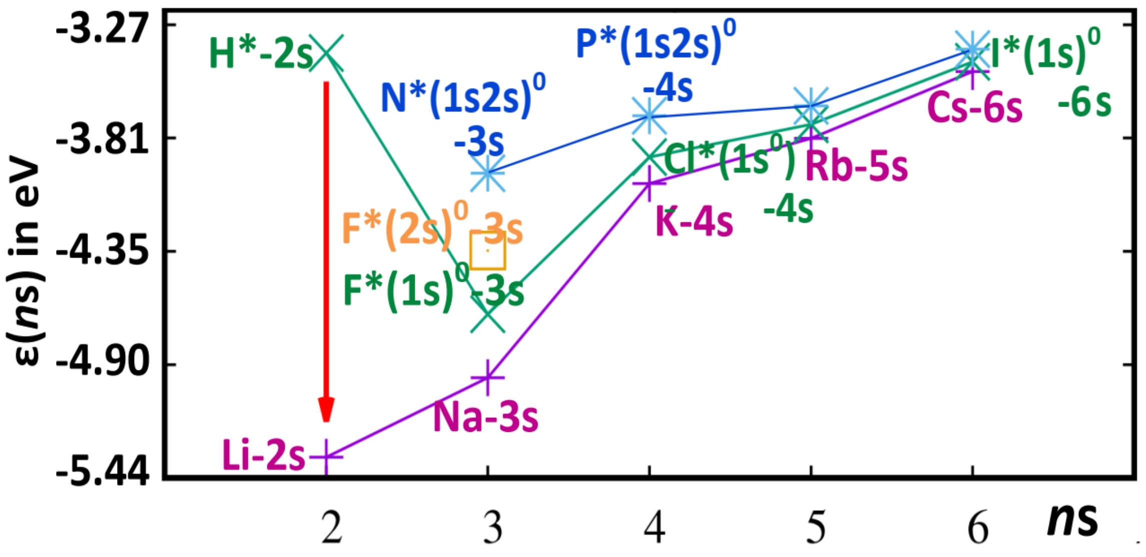


***Figure S3.*** Energies ε (in eV) of atomic *n*s valence orbitals (*n* = 2 to 6) from numerical Dirac-Fock self-consistent field calculations. **Lilac**: electronic ground-states of alkali-metal atoms (Li-2s to Cs-6s) with nuclear charge number $Z_o$ and filled core shells. **Green**: core-excited states of atoms with $Z = Z_o-2$ and empty $1s^0$ shell (H*-$2s^1$, F*-$1s^02s^22p^63s^1$, Cl*-$1s^02s^2\cdots3p^64s^1$, Br*-$1s^02s^2\cdots4p^65s^1$, I*-$1s^02s^2\cdots5p^66s^1$). **Yellow** single box: excited F*-$1s^22s^02p^6$-3s with $Z = Z_o-2$ and empty $2s^0$ core shell. **Blue**: highly excited states of atoms with $Z = Z_o-4$ and empty $1s^0$ $2s^0$ core shells (N*-$1s^02s^02p^63s^1$, P*-$1s^02s^02p^63s^23p^64s^1$, As*-$1s^02s^02p^63s^23p^63d^{10}4s^24p^65s^1$, Sb*-$1s^02s^02p^6\cdots5p^66s^1$).

## S.8 Additional References

[S1] R. Bader, *Atoms in Molecules: A Quantum Theory*. Oxford University Press, **1994**.

[S2] F. M. Bickelhaupt, E. J. Baerends, *Rev. Comput. Chem.* **2000,** 15, 1.

[S3] F. Weinhold, C. Landis, *Valency and Bonding: A Natural Bond Orbital Donor–Acceptor Perspective*. Cambridge University Press, **2005**.

[S4] K. Ruedenberg, M. W. Schmidt, *J. Comput. Chem.* **2007**, 28, 391.

[S5] A. C. West, M. W. Schmidt, M. S. Gordon, K. Ruedenberg, *J. Chem. Phys.* **2013**, 139, 234107.

[S6] M. W. Schmidt, J. Ivanic, J., K. Ruedenberg, *J. Chem. Phys.* **2014**, 140, 204104.

[S7] P. Pyykkö, H. Stoll, in A. Hinchliffe (ed.) *Chemical Modelling, Applications and Theory*, Vol. 1, Ch. 5, p. 239. Royal Society of Chemistry, Cambridge GB, **2000**.

[S8] ADF 2017.101, SCM, Theoretical Chemistry, Vrije Universiteit, Amsterdam, **2017**. (http://www.scm.com).

[S9] M. LeBellac, J.-M. Lévy-Leblond, Galilean Electromagnetism, *Nuovo Cim. B* **1973**, *14*, 217.

[S10] N. Bohr, *Philos. Mag. Ser. 6*, **1913**, *26*, 1; *Nature*, **1913**, *92*, 231.

[S11] A. Sommerfeld, *Sitzungsber. der Münchener Akad. Wiss.*, **1915**, 425 & 459; *Ann. Physik IV*, **1916**, 51, 1.

[S12] R. E. Langer, *Phys. Rev.* **1937**, 51, 669.

[S13] R. P. Feynman, *Rev. Mod. Phys.* **1948***, 20,* 367.

[S14] M. V. Berry, K. E. Mount, *Rep. Prog. Phys.*, **1972**, 35, 315.

[S15] T. A Heim, *J. Phys. B*, **1994**, 27, 225.

[S16] H. Friedrich, J. Trost, *Phys. Rev. Let.*, **1996**, 76, 4869.

[S17] J. B. Bronzan, *Phys. Rev. A*, **1996**, 54, 41.

[S18] J. Hainz, H. Grabert, *Phys. Rev. A*, **1999**, 60, 1698.

[S19] X.-Y. Gu, S.-H. Dong, *Phys. Let. A*, **2008**, 372, 1972.

[S20] B.-F. Li, T. Zhu, A.-z. Wang, *Universe*, **2020**, 6, 90.

[S21] D. P. Wong, *J. Chem. Educ.* **1979**, *56*, 714.

[S22] H. A. Bent, F. Weinhold, *J. Chem. Educ.* **2007**, *84*, 1145.

[S23] E. R. Scerri, *J. Chem. Educ.* **1998**, *75*, 1384.

[S24] R. C. Millikan, *J. Chem. Educ.* **1982**, *59*, 757.

[S25] W. H. E. Schwarz, R. L. Rich, *J. Chem. Educ.* **2001**, *87*, 435.

[S26] J. P. Connerade. *Contemp. Phys.* **1978**, *19*, 415; *J. Phys. B* **1978**, *24*, L109.

[S27] J. P. Perdew, K. Burke, M. Ernzerhof, *Phys. Rev. Lett.* **1996**, *77*, 3865.

[S28] E. van Lenthe, E. J. Baerends, *J. Comput. Chem.* **2003**, *24*, 1142.
